# The protective effect of *Satureja bachtiarica* hydroalcoholic extract on streptozotocin‐induced diabetes through modulating glucose transporter 2 and 4 expression and inhibiting oxidative stress

**DOI:** 10.1080/13880209.2019.1597131

**Published:** 2019-05-07

**Authors:** Reyhaneh Joudaki, Mahbubeh Setorki

**Affiliations:** Department of Biology, Izeh Branch, Islamic Azad University, Izeh, Iran

**Keywords:** *Satureja bachtiarica*, diabetes, streptozotocine, oxidative stress, glucose transporter

## Abstract

**Context:** Oxidative stress plays an important role in development of diabetes mellitus. *Satureja bachtiarica* Bunge (Lamiaceae) is a rich source of bioactive compounds with antioxidant properties.

**Objective:** This study investigates the antidiabetic effect of hydroalcoholic extract of aerial parts of *S. bachtiarica*.

**Methods and materials:** Male Wistar rats were randomly divided into six groups (*n* = 8) including control (normal saline), diabetic [Streptozotocin (STZ)], intervention (STZ plus hydroalcoholic extract of *S. bachtiarica* at doses of 75, 150 and 250 mg/kg/d) and positive control (STZ plus captopril 50 mg/kg/d) groups. A single intraperitoneal (IP) injection of STZ (60 mg/kg) was used to induce diabetes and IP therapy with drugs was performed for four weeks.

**Results**: In diabetic rats, serum total antioxidant capacity (TAC) decreased significantly, but glucose, alkaline phosphatase *(*ALP), alanine aminotransferase (ALT), aspartate aminotransferase (AST), γ-glutamyltransferase (GGT) and malondialdehyde (MDA) increased significantly as compared to the control (*p* < 0.05). Treatment with extract (250 mg/kg) caused a significant decline in serum glucose, GGT, ALT, AST and MDA as well as a significant increase in serum TAC (*p* < 0.05). During the intervention period, diabetic rats showed significant weight loss, but extract (250 mg/kg) treated rats did not show any weight loss. Extract (250 mg/kg) up-regulated *GLUT2* expression and down-regulated *GLUT4* expression in the liver (*p* < 0.05). *S*. *bachtiarica* extract at all dosage levels prevented STZ-induced histological damage of liver, kidney and pancreas.

**Discussion and conclusions:***S*. *bachtiarica* extract exhibits antidiabetic effects through modulation of oxidative stress and expression of *GLUT2* and *GLUT4*.

## Introduction

Diabetes is one of the most common metabolic disorders of the current era. The global prevalence of diabetes was estimated to be 8.8% in 2015 and predicted to rise to 10.4% in 2040 (Ogurtsova et al. 2017). In diabetes, the body loses its ability to produce insulin hormone or becomes resistant to the action of insulin, and, therefore, the produced insulin cannot function properly. As a result, the body’s function to use and metabolize glucose is demonstrably reduced and blood glucose level increases, which known as hyperglycaemia (American Diabetes Association [ADA] 2017). Chronic hyperglycaemia can lead to the damage of small blood vessels and subsequent failure of various organs, such as kidneys, eyes and nerves (Abouzed et al. 2018). Diabetes is also directly correlated with the increased risk of developing cardiovascular disease (Mahmoud et al. 2017).

Diabetes has two main types, in type 1 diabetes which comprises 5–10% of all diagnosed diabetes cases, the autoimmune destruction of pancreatic β-cells leads to the defective insulin secretion. In type 2 diabetes which accounts for 90–95% of cases of diabetes, the body’s cells are becoming progressively resistant to the action of insulin, which may ultimately lead to the destruction of pancreatic β-cells and complete impairment of insulin production (Oyenihi et al. 2017). Genetic factors, obesity and lack of physical activity are known to play an important role in the development of type 2 diabetes (Mahmoud et al. 2017).

Impairment of insulin signalling in skeletal muscle, liver and adipose tissues due to the progressive insulin resistance is the main cause of type 2 diabetes. Certain biochemical factors, such as inflammatory markers and adipokines, which are usually secreted from the liver and adipose tissues, are also involved in the development of type 2 diabetes (Oyenihi et al. 2017).

Hyperglycaemia and oxidative stress play an important role in the aetiology and pathology of diabetic complications. Chronic hyperglycaemia induces oxidative stress and increases the production of reactive oxygen species (ROS) and free radicals (Oyenihi et al. 2017). Several hypotheses have been proposed to explain the mechanisms of increased ROS generation during diabetes, including glucose autoxidation, non-enzymatic and progressive protein glycosylation, increased glycine end-product and polyol pathway (Sadek and Shaheen 2014). ROS, such as hydroxyl and superoxide radicals attack important cellular macromolecules, such as carbohydrates, nucleic acids, lipids and protein leading to cell damage and death (Sadek et al. 2017).

Type 2 diabetes is also associated with a variety of lipid disorders, and evidence suggests that hyperlipidaemia, especially hypercholesterolemia, plays a key role in the development of cardiovascular diseases and atherosclerosis in diabetic patients (Xu et al. 2019). The severity of lipid disorders in diabetic patients depends on the level of insulin secretion, insulin resistance, obesity, diet and the presence of underlying or secondary causes of hyperlipidaemia. Hyperlipidaemia exacerbates hyperglycaemia-induced oxidative stress and related tissue damage through increasing serum and tissues free fatty acids (FFA) (Xu et al. 2019).

The facilitated transport of glucose from the plasma membrane of the mammalian tissues is catalysed by a family of glucose transporters (GLUTs). GLUT2 isoform is found abundantly in the liver and pancreatic β-cells, and GLUT4 isoform is found in the skeletal muscle cells and adipocytes (Jung et al. 2006). Certain therapeutic interventions affecting these transporters have been shown to improve glucose and insulin metabolism and ultimately prevent and manage diabetic disorders (Zygmunt et al. 2010).

Regarding the role of oxidative stress and inflammation in the pathogenesis of type 2 diabetes, the researchers are now focused on the use of natural compounds with antioxidant, anti-inflammatory and hepatoprotective effects to manage diabetic complications (Coskun et al. 2005). *Satureja bachtiarica* Bunge (Lamiaceae) is widely distributed in Iran and has been reported to grow in western, central and southwestern provinces of the country (Soodi et al. 2016). In Iranian traditional medicine*, S*. *bachtiarica* is used as a carminative, appetizer and sexual enhancer. *S. bachtiarica* is also recommended for the treatment of cough, dyspnoea, diarrhoea and stomach cramps (Khadivi-Khub et al. 2015). Pharmacological studies have demonstrated antioxidant (Soodi et al. 2016), anticancer (Behdarvand Shoushtar and Ghasemi Pirbaloti 2017), neuroprotective (Soodi et al. 2012), anti-inflammatory and analgesic (Saghaei and Motamedi 2017) properties of *S*. *bachtiarica* extract. Therefore, this study investigates the efficacy of *S*. *bachtiarica* extract on STZ-induced hyperglycaemia, oxidative stress, tissue damage and expression of *GLUT2* and *GLUT4* receptors in diabetic rats.

## Materials and methods

### Chemicals

2,2-Diphenyl-1-picrylhydrazyl (DPPH), 2,2′-azinobis (3-ethylbenzothiazoline-6-sulfonic acid) (ABTS) and 3-(2-pyridyl)-5,6-diphenyl-1,2,4-triazine-4′,4″-disulphonic acid sodium salt (Ferrozine) were purchased from Sigma-Aldrich Chemical Co., St Louis, MO. Other chemicals were laboratory reagent grade and obtained from Merck Co. (Darmstadt, Germany).

### Preparation of hydroalcoholic extract

Aerial parts of *S. bachtiarica* were collected from Khuzestan Province during summer 2017, and after identification by Dr. Rafieian*-*Kopaei, botanist, a specimen voucher (no: 987,689) was deposited at the Herbarium of Islamic Azad University of Izeh, Khuzestan. *S*. *bachtiarica* aerial parts were shade dried at room temperature and then pulverized with an electric mill. The extraction was carried out by using the maceration process. The plant powder was mixed with water and ethanol (70:30 v/v) and stored at room temperature in the dark for 48 h. Then, the resulting mixture was filtered using a filter paper, concentrated using a rotary evaporator, and then incubated at 37 °C to until complete dryness (Sowndhararajan and Kang 2013).

### Evaluation of *in vitro* antioxidant effects

#### DPPH radical scavenging activity

Different concentrations of extract were prepared in distilled water, and 1 mL of resulting solutions were mixed with 1 mL of 0.1 mM DPPH solution (prepared in 95% ethanol) and allowed to stand for 15 min at room temperature and then, the absorbance of the samples were recorded at 517 nm using a spectrophotometer. The control was prepared using 1 mL of distilled water instead of the sample. The percentage of DPPH radicals scavenging activity was determined using the following formula: %DPPH radical scavenging activity = [(A_control_ – A_sample_)/A_control_] × 100. IC_50_ value obtained by plotting a graph of concentration (X-axis) *versus* the percentage of inhibition (Y-axis) (Sowndhararajan and Kang 2013).

#### Fe^2+^ chelating activity

First, extract solution (1 mL) at different concentrations was mixed with 3.7 mL of distilled water. Then, 0.1 mL of 2 mM FeCl_2_ and 0.2 mL of 5 mM ferrozine were added to the mixture. After approximately 20 min, the optical absorbance was read at 562 nm. For the control sample, distilled water was used instead of the sample. Fe^2+^ chelating activity was calculated using the following formula. Fe^2+^ chelating activity (%) = [(A_control_ – A_sample_)/A_control_] × 100. IC_50_ value was calculated from the plot of the chelating activity against the sample concentrations (Sowndhararajan and Kang 2013).

#### ABTS radical scavenging activity

ABTS working solution was prepared by reacting ABTS (7.4 mM, 10 mL) with potassium persulfate (2.6 mM, 10 mL) for 12 h at room temperature in the dark. Before the experiment, freshly prepared ABTS solution was diluted with methanol to reach an absorbance of 1.1 ± 0.02 at 734 nm. Then, 150 μL of the extract at different concentrations was added to 2850 μL of ABTS solution, and after incubation at ambient temperature for 12 h, the optical absorbance was recorded. The control sample was prepared using 150 μL of distilled water instead of the sample. ABTS scavenging activity was determined using the following formula. ABTS scavenging activity (%) = [(A_control_ – A_sample_)/A_Control_] × 100. IC_50_ value was determined from the plot of the scavenging activity against the sample concentrations (Sowndhararajan and Kang 2013).

#### Hydroxyl radical scavenging activity

First, 1, 10-phenanthroline solution (1.865 mM, 1 mL) and the extract at different concentration (2 mL) were added into a test tube and mixed well. Then, mL of the FeSO_4_ solution (1.865 mM) was added to the mixture. The reaction was started by adding 1 mL of H_2_O_2_ (3% v/v) and after 60 min of incubation in a 37 °C water bath, the absorbance was recorded at 536 nm. The solution containing extract without hydrogen peroxide was considered as the blank and solution without extract was considered as the negative control. Hydroxyl radical scavenging activity was determined using the following formula. Hydroxyl radical scavenging activity (%) = [(As-An)/(Ab-An)] × 100; where As is the absorbance of the sample, An is the absorbance of the negative control and Ab is the absorbance of the blank. IC_50_ value was determined from the plot of the scavenging activity against the sample concentration (Sowndhararajan and Kang 2013).

#### Laboratory animals

Male Wistar rats weighing 250–300 g were used in this study. Animals were kept in the Animal House of the Islamic Azad University of Izeh, Khuzestan at the appropriate temperature (21 ± 2 °C) and 12 h light/dark cycle with free access to water and food. All animal procedure was based on Guidelines for the Care and Use of Laboratory Animals (Institute for Laboratory Animal Research 1985).

#### Diabetes induction and animal grouping

The animals with a fasting blood glucose level of less than 130 mg/mL were used in this study. Intraperitoneal (IP) injection of a single dose of streptozotocin (STZ) (60 mg/kg) was used to induce diabetes in rats. After observing the symptoms of diabetes within 72 h (weight loss, increase in the blood glucose level (over 250 mg/mL) and polydipsia), treatment using the extract or captopril was performed for four weeks. Rats were divided randomly into six groups (*n* = 8) including a group of healthy rats (treated with normal saline, IP), a group of diabetic rats (treated with normal saline, IP), 3 groups of diabetic rats treated with *S. bachtiarica* extract at doses of 75, 150 and 250 mg/kg using IP injection and a groups of diabetic rats treated with captopril at a dose of 50 mg/kg using IP injection. After four weeks of treatment, rats were fasted for 12 h and then anesthetized with chloroform. Blood samples were collected from the heart of animals and centrifuged to separate the sera. Sera were immediately stored at −70 °C until biochemical analysis. Animals were then autopsied, and the liver, pancreas and kidney were removed and fixed in 10% formalin for pathophysiological analysis. The animal weight was measured by using a digital scale before and after treatment (Dewanjee et al. 2009).

#### Serum biochemical factors

The serum levels of lipids [total triglyceride (TG), total cholesterol (TC) and high-density lipoprotein cholesterol (HDL-C)], liver enzymes [aspartate aminotransferase (AST), alkaline phosphatase (ALP), alanine aminotransferase (ALT) and γ-glutamyltransferase (GGT)], as well as urea, creatinine and albumin were measured by commercial kits (Pars Azmoon Co., Tehran, Iran) using an autoanalyzer (Biotecnica BT-3000, Tokyo, Japan) (Dewanjee et al. 2009).

#### Serum malondialdehyde (MDA) assay

Briefly, 1.5 mL of 20% acetic acid, 1.5 mL of 0.8% thiobarbituric acid (TBA), 200 μL of 1.8% sodium dodecyl sulphate (SDS) and 700 μL of distilled water were added to the test tubes containing 100 μL of serum samples. The tubes were then immersed in a boiling water bath for 60 min, then 1 mL of distilled water and 5 mL of butanol/pyridine were added to the samples, and the samples were centrifuged and the optical absorbance of the supernatant was recorded at 532 nm (Dewanjee et al. 2009).

#### Total antioxidant capacity (TAC) measurement

Ferric reducing power (FRAP) assay was used to measure the serum TAC. The FRAP solution was prepared by adding 10 mL of acetate buffer (0.25 M, pH = 3.6), 5 mL of TPTZ (10 mM) [prepared in HCl (40 mM)], and 2.5 mL of 6H_2_OFeCl_3_ (20 mM). 25 μL of the serum was mixed with 1.5 mL of the FRAP solution, and the resulting mixture was left at 37 °C for 10 min and then the optical absorbance at 593 nm was recorded (Dewanjee et al. 2009).

#### Histological studies

The pancreas, liver and kidney tissues were fixed in 10% buffered formalin solution, processed and embedded in paraffin and 3–5 μm sections were prepared and placed on the glass slides. The slides were then stained with haematoxylin-eosin (H & E) and used for histological evaluation under a light microscope (Dewanjee et al. 2009).

## Measurement of *GLUT2* and *GLUT4* gene expression

Samples of rat liver were frozen in an adequate volume of acid guanidinium thiocyanate solution and kept at −80 °C until RNA extraction. Total cellular RNA was extracted by the method of acid guanidinium thiocyanate phenol/chloroform extraction. The total tissue RNA concentration was measured by spectrophotometric absorbance (260 nm), and the quality of isolated RNA was verified by agarose gel electrophoresis with ethidium bromide staining. Purified total RNA (1 µg) was used as a substrate for reverse transcription. The reaction was performed by incubation of RNA with 1 µM oligo (dT) and 200 units of MMLV reverse transcriptase from a Clontech (Mountain View, CA) first strand cDNA synthesis kit. An aliquot (5 µL of a 1/10 dilution) of the cDNA of each sample was used for RT-PCR. The PCR primers used are shown in Table 1. DNA amplification was carried out in 1 × Taq polymerase buffer, 1.5 mM MgCI_2_ supplemented with 50 µM dNTP, 0.25 µM of 5′ and 3′-specific primers, 1 µCi of [α-32p] and 2 units of Taq polymerase (Promega C) in a final volume of 50 µL. The mixture was overlaid with mineral oil and amplified for 30 cycles (each consisting of denaturation for 1 min at 94 °C, annealing for 1 min at 60 °C, extension for 1 min at 72 °C) then extension for 7 min at 72 °C and storage at 4 °C in a Triothermoblock. cDNA products (10 µL) were size-separated by electrophoresis on a 10% acryl/bisacrylamide gel and stained with ethidium bromide (15 µg/mL). Each band was excised from the gel, and the quantity of 32p incorporated was measured in a scintillation counter (Weinstein et al. 1994).

**Table 1.** Primers for *GLUT2*, *GLUT4* and *GAPDH* gene.

Genes	Forward	Reverse
*GAPDH* (control gen)	GTA TTG GGC GCC TGG TCA CC	CGC TCC TGG AAG ATG GTG ATG G
*GLUT4*	ACATACCTGACAGGGCAAGG	CGCCCTTAGTTGGTCAGAAG
*GLUT2*	GGCTAATTTCAGGACTGGTT	TTTCTTTGCCCTGACTTCCT

### Data analysis

Data were analysed using SPSS version 20 (SPSS Inc., Chicago, IL). Analysis of variance (ANOVA) followed by Duncan’s test used to identify statistical differences between means. All data were presented as mean ± SD and *p* value less than 0.05 was considered statistically significant.

## Results

### Results of *in vitro* antioxidant assays

In this study, the antioxidant activity of *S*. *bachtiarica* extract was evaluated *in vitro* by using five antioxidant tests. The results of *in vitro* assays showed that *S*. *bachtiarica* extract had strong DPPH radical scavenging activity (IC_50_ = 67.02 µg/mL), moderate ABTS and hydroxyl radicals scavenging activity (IC_50_ = 468.52 and 303.56 µg/mL, respectively) and relatively good ferrous iron chelating activity (IC_50_ = 1639.38 µg/mL), and strong ferric reducing activity (optical absorbance at 700 nm = 2.7).

### Results of laboratory animals

**Figure 1.**
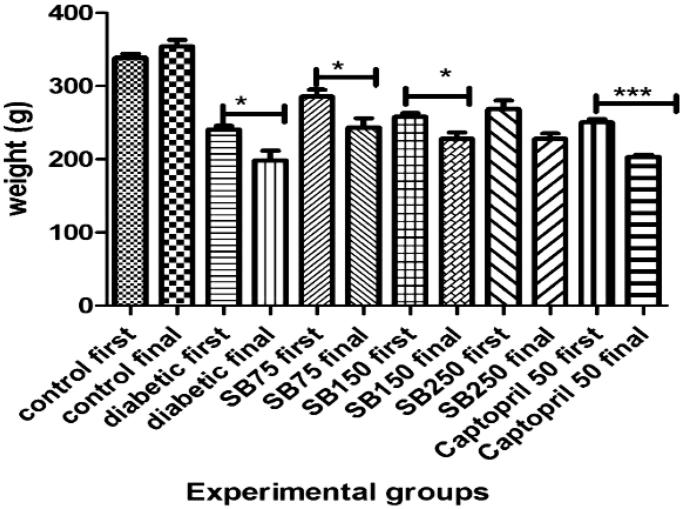
Comparison of body weight change during the study period between the studied groups (SB: *Satureja bachtiarica*); *Significant difference compared to values before treatment (***p < 0.001 and *< 0.05).

According to the results, STZ-treated rats exhibited significant weight loss over the 4 week period (*p* < 0.05), while there was no significant change in the weight of the control group. The weight of diabetic rats treated with *S*. *bachtiarica* extract at doses of 75 and 150 mg/kg also showed a significant reduction (*p* < 0.05), but, rats treated with extract at a dose of 250 mg/kg did not show any significant weight loss (Figure 1).

**Figure 2.**
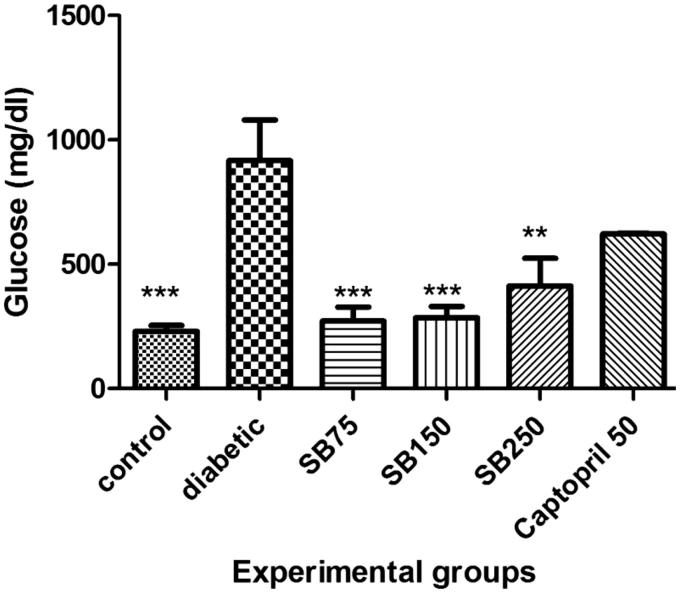
Comparison of blood glucose levels between the studied groups (SB: *Satureja Bacchitaria*); *Show significant difference compared to the diabetic group (***p < 0.001 and **< 0.01).

The blood glucose level in the diabetic group was significantly higher than that in the control group (*p* < 0.001). Administration of *S*. *bachtiarica* extract at doses of 75, 150, and 250 mg/kg to diabetic rats produced a significant decrease in the blood glucose level compared to the diabetic group (*p* < 0.001 and < 0.001). Captopril treatment also reduced blood glucose level compared to the diabetic rats, but the difference was not statistically significant (Figure 2).

**Figure 3.**
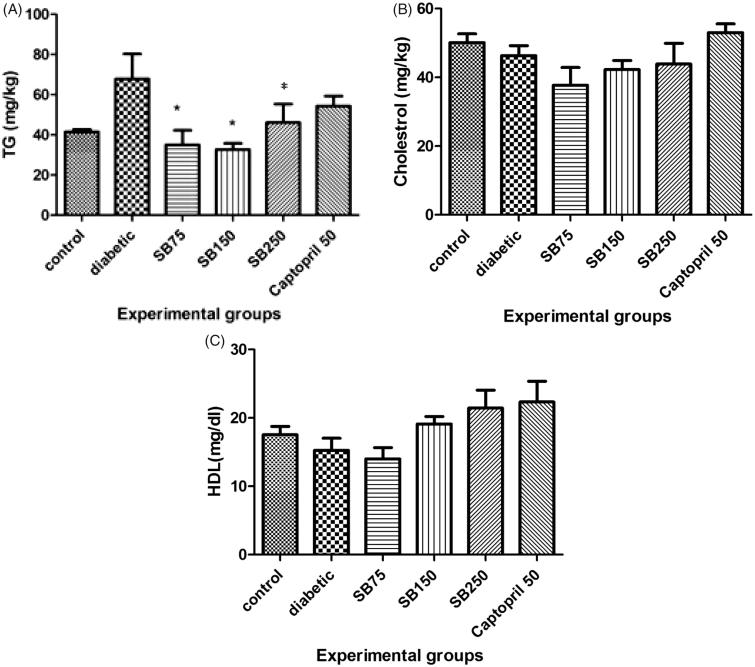
Comparison of serum triglyceride (TG) (A), total cholesterol (TC) (B), and high-density lipoprotein-cholesterol (HDL-C) (C) levels between the studied groups (SB: *Satureja bachtiarica*). In Figure A: *Show significant difference compared to the diabetic group (*p < 0.05).

As shown in Figure 3, the serum TG level increased in diabetic rats as compared to the control group, but the increase was not statistically significant. Administration of *S*. *bachtiarica* extracts (75, 150 and 250 mg/kg) to diabetic rats significantly decreased blood TG level when compared to the diabetic group (*p* < 0.05, Figure 3). There were no significant differences in serum TC and HDL-C levels between the studied groups (Figure 3(B,C)).

**Figure 4.**
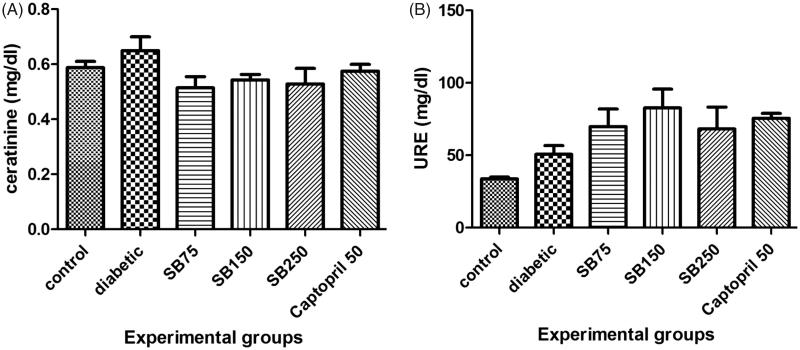
Comparison of serum creatinine (A) and urea (B) levels between the studied groups (SB: *Satureja bachtiarica*).

**Figure 5.**
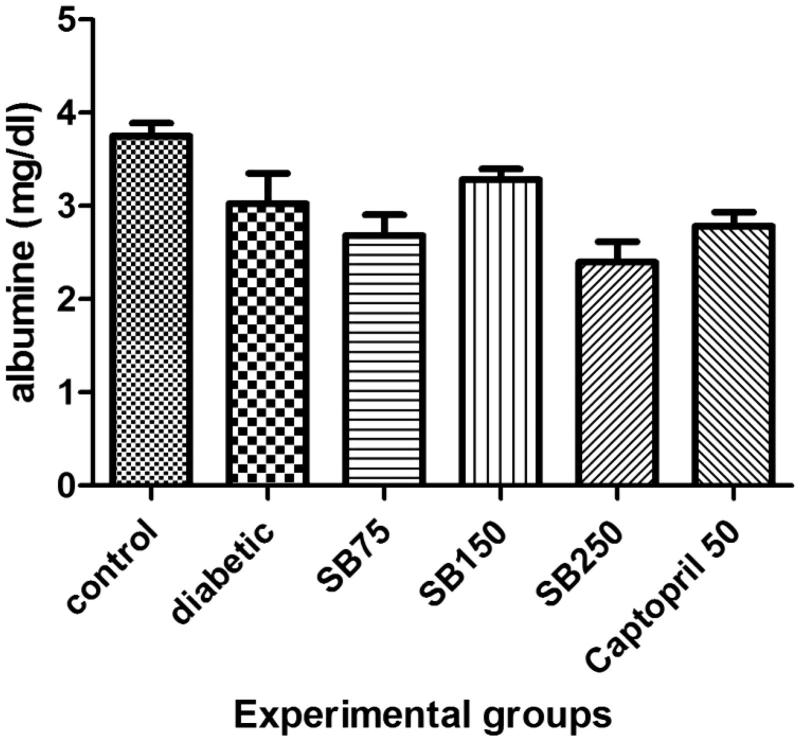
Comparison of serum albumin level between the studied groups (SB: *Satureja bachtiarica*).

Serum urea and creatinine levels in healthy, diabetic and diabetic rats treated with extract or captopril showed no statistically significant differences (Figure 4(A,B)). Serum albumin level also showed no significant difference between the groups (Figure 5).

**Figure 6.**
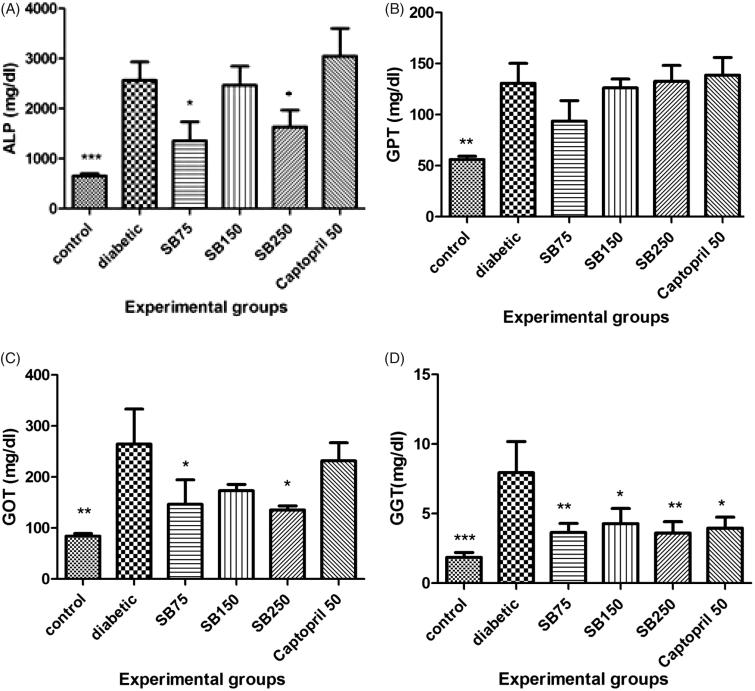
Comparison of alkaline phosphatase (A), alanine aminotransferase (B), aspartate aminotransferase (C) and gamma glutamyltransferase (D) between the studied groups (SB: *Satureja bachtiarica)*. *Show significant difference compared to the diabetic group (***p < 0.001, **< 0.01 and *< 0.05).

STZ-treated rats showed a significant elevation in the serum levels of ALP, ALT, AST and GGT as compared to the control group (*p* < 0.001 and *p* < 0.01). Administration of *S*. *bachtiarica* extract at 75 and 250 mg/kg significantly decreased serum ALP (*p* < 0.05) and AST (*p* < 0.05) levels and at 75, 150 and 250 mg/kg, significantly decreased serum GGT level (*p* < 0.05 and 0.01, Figure 6(A*–*D)).

**Figure 7.**
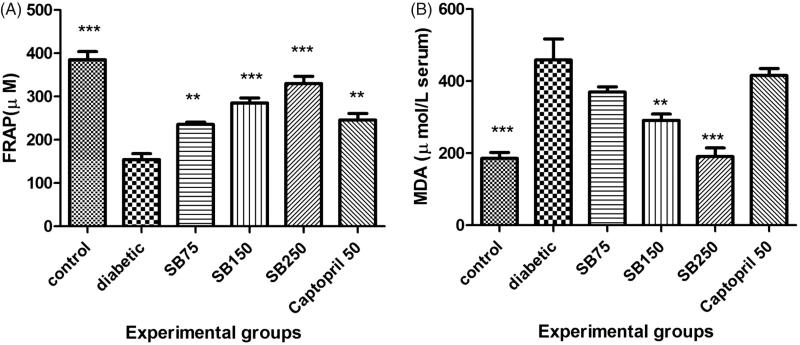
Comparison of serum total antioxidant capacity (A) and malondialdehyde level (B) among the studied groups (SB: *Satureja bachtiarica*). *Show significant difference compared to diabetic group (***p < 0.001 and *< 0.05).

STZ-induced diabetic rats also showed a significant reduction of serum TAC and a significant increase of serum MDA when compared to the healthy rats (*p* < 0.001, Figure 6(A,B)). Treatment of diabetic rats with *S*. *bachtiarica* extract at doses of 75, 150 and 250 mg/kg considerably increased serum TAC (*p* < 0.01 and < 0.001), and at doses of 150 and 250 mg/kg, significantly decreased serum MDA level (*p* < 0.01 and < 0.001, Figure 7(A,B)).

Treatment of diabetic rats with *S*. *bachtiarica* extract at doses of 150 and 250 mg/kg caused a significant decrease and at a dose of 75 mg/kg caused a significant increase in the expression of *GLUT2*. Also, *S*. *bachtiarica* extract at 250 and 75 mg/kg increased and at 150 mg/kg, decreased the expression of *GLUT4* (Table 2).

**Table 2.** Comparison of *GLUT2* and *GLUT4* gene expression between studied groups.

Groups	*GLUT4*	*GLUT2*
Diabetic	1.00 ± 0.22	1.00 ± 0.27
SB75	4.85 ± 1.28*	1.96 ± 0.48
SB150	0.44 ± 0.03*	0.06 ± 0.02*
SB250	1.79 ± 0.34*	0.10 ± 0.08*
Captopril	0.10 ± 0.02	0.03 ± 0.00

*Significant difference compared to diabetic group (*< 0.05).

**Image 1.**
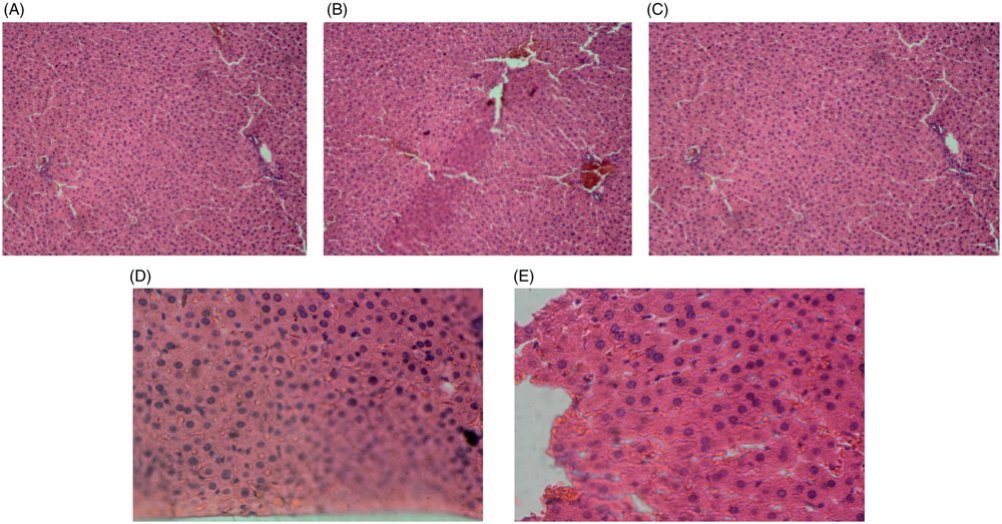
Liver tissue sections of control rats (A), diabetic rats (B), diabetic rats treated with 75 mg/kg of *S. bachtiarica* extract (C), diabetic rats treated with 150 mg/kg of *S*. *bachtiarica* extract (D) and diabetic rats treated with 250 mg/kg of *S*. *bachtiarica* extract (E); haematoxylin-eosin staining; magnification x40.

As histological examination shows, the liver tissue of the control group had a completely normal histological structure. In diabetic rats, severe hyperaemia and destruction of hepatic lobule, hepatocytes and sinusoids were observed. In diabetic rats treated with different doses of extract, the sinusoids, hepatocytes, hepatic lobule, central veins and portal triads were completely normal (Image 1).

**Image 2.**
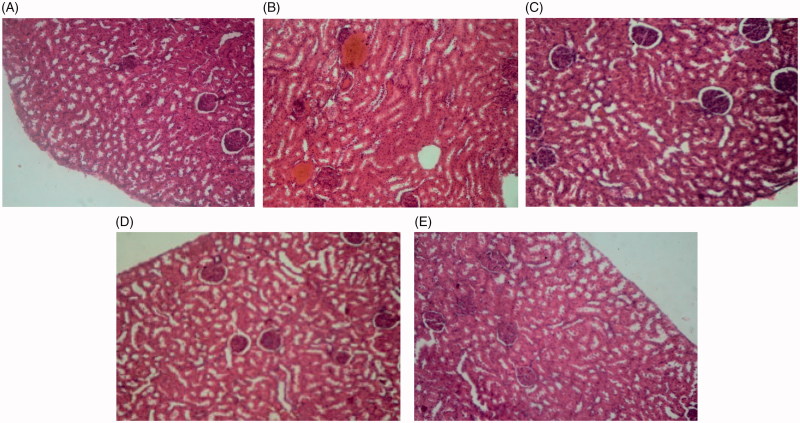
Renal tissue sections of control rats (A), diabetic rats (B), diabetic rats treated with 75 mg/kg of *S. bachtiarica* extract (C), diabetic rats treated with 150 mg/kg of *S*. *bachtiarica* extract (D) and diabetic rats treated with 250 mg/kg of *S*. *bachtiarica* extract (E); haematoxylin-eosin staining; magnification x40.

In control rats, nephron, as well as proximal and distal tubules, had normal structure. In the diabetic group, destruction of the renal tissue in the cortex and complete degeneration of the nephrons were observed. In diabetic rats treated with *S*. *bachtiarica* extract, tubules of the nephron, renal corpuscle, Bowman’s capsule were healthy and normal (Image 2).

**Image 3.**
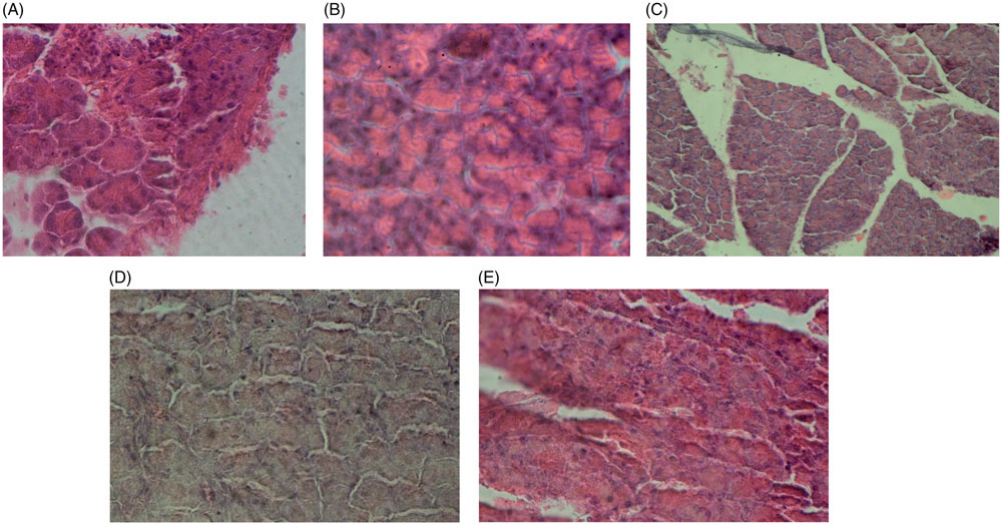
Pancreas tissue sections of control rats (A), diabetic rats (B), diabetic rats treated with 75 mg/kg of *S. bachtiarica* extract (C), diabetic rats treated with 150 mg/kg of *S*. *bachtiarica* extract (D) and diabetic rats treated with 250 mg/kg of *S*. *bachtiarica* extract (E); haematoxylin-eosin staining; magnification x40.

As Image 3 shows, in non-diabetic healthy rats, the pancreatic acinar cells and islets of Langerhans were healthy and had a normal appearance. In the diabetic group, hyperaemia and the acidophilia of the acinar cells were observed. In diabetic rats treated with *S*. *bachtiarica* extract, the acinar cells and islets of Langerhans were normal and healthy.

## Discussion

This study investigated the protective effects of *S*. *bachtiarica* extract on STZ-induced diabetes in rats. It was observed that the induction of diabetes in rat causes a significant increase in blood glucose and MDA and a significant decrease in serum TAC.

STZ, an antibiotic produced by *Streptomyces achromogenes*, is typically used to induce diabetes in experimental models (Abouzed et al. 2018). STZ is taken up by the pancreatic β-cells through the GLUT2 glucose transporter and causes degenerative changes in the cells which leads to the decreased insulin secretion and ultimately hyperglycaemia (Eleazu et al. 2013). The mechanism of STZ action on pancreatic β-cells has not yet been fully understood; however, it is believed that oxidative stress plays an important role in the development of the STZ-induced complications (Sadek et al. 2017). STZ is an unstable molecule that accumulates in the pancreatic β-cells and degrades to carbonium radicals. Carbonium radicals are highly reactive and induce direct toxic effects on pancreatic islet cell; they also show indirect toxic effects by increasing the generation of ROS (Ghanema and Sadek 2012; Sadek et al. 2017). STZ was reported to cause β-cell damage through the induction of DNA fragmentation and methylation (Cardinal et al. 2001). It has also been shown that STZ causes inflammatory response through enhanced infiltration of mononuclear cells and excessive production of inflammatory cytokines in the islets of Langerhans that eventually leads to the β-cells injury and death (Abouzed et al. 2018). Also, studies have shown that increased nitric oxide (NO) production in the pancreas of STZ-treated rats plays an important role in the degeneration of β-cells (Pacher et al. 2005). In STZ-treated rats, serum NO level was reported to be elevated (Coskun et al. 2005), and inhibitors of nitric oxide synthases (NOS) such as LNG-NMDA and aminoguanidine resulted in remarkably reduced hyperglycaemia and β-cells damage (Lukic et al. 1991; Corbett and McDaniel 1996). In our study, hyperglycaemia, lipid peroxidation and decreased serum TAC in STZ-diabetic rats confirmed these mechanisms.

In diabetic rats, treatment with *S*. *bachtiarica* extract (150 and 250 mg/kg) improved hyperglycaemia, decreased lipid peroxidation and increased serum TAC. In previous studies, the antioxidant effects of *S*. *bachtiarica* have been observed both *in vitro* (Hashemi et al. 2011) and *in vivo* (Soodi et al. 2016) which are in harmony with our study. In this study, *S*. *bachtiarica* extract significantly ameliorated histopathological damage of pancreas in STZ-treated rats, which confirms its protective effects on pancreatic β cells.

The weight of diabetic rats significantly decreased after 4 weeks of treatment, while there was no significant change in weight of control rats and diabetic rats treated with 250 mg/kg of *S*. *bachtiarica* extract. Loss of body weight in diabetic rats may be due to the proteolytic degradation of structural proteins into amino acids that are eventually oxidized and used as a source of energy because the body’s cells are unable to absorb blood glucose as a source of metabolic energy. Amino acids may also be used as gluconeogenic precursors in the liver (Sathish Sekar et al. 2005), Also, glycogenolysis and lipolysis play a role in the weight loss of diabetic rats (Dewanjee et al. 2009). The ability of *S*. *bachtiarica* extract (250 mg/kg) to prevent the weight loss of diabetic rats can be due to its beneficial effects in reducing blood glucose.

Studies have reported that STZ-induced diabetes is associated with lipid disorders (increased TG, TC, LDL and VLDL, as well as decreased HDL) (Almeida et al. 2012). Several biochemical mechanisms have been proposed for lipid disorders due to diabetes. Following the induction of diabetes, the activity of hormone-sensitive lipases increases, which results in the breakdown of triglycerides stored in adipocytes into fatty acids and their release into the blood. It has also been reported that the activity of lipoprotein lipase (endothelium-bound glycoprotein), which plays an important role in the degradation of triacylglycerol, chylomicrons and VLDL, is reduced following induction of diabetes (Almeida et al. 2012). In our study, STZ-treated rats demonstrated an insignificant and minor increase in serum TG and also an insignificant decrease in serum HDL, treatment with of *S*. *bachtiarica* extract at all doses significantly reduced serum TG when compared to the diabetic rats.

In this study, significant increases in serum ALP, ALT, AST and GGT were observed in STZ-treated rats, but no significant changes were observed in the serum levels of creatinine, urea and albumin. STZ exhibits various biological activities including toxicity, carcinogenicity, teratogenesis and mutagenesis, and not only damages pancreatic beta cells, but also produces toxic effects in other tissues and organs of the body, such as the kidney, liver and gastrointestinal tract (Quine and Raghu 2005; El Rabey et al. 2017; Abouzed et al. 2018). In this study, the results of the histopathological examination showed that STZ injection causes tissue damage in not only pancreas but also liver and kidney. Increased blood levels of liver enzymes also confirmed histological damage of the liver. However, we did not observe any increase in the biochemical markers of renal injury. Studies have reported that STZ-induced damage to liver, kidney and gastrointestinal tract increase dramatically from the time of injection to six weeks later (Piyachaturawat et al. 1988; Zafar et al. 2009). So, the lack of a significant change in serum creatinine and urea in our study may be related to the shorter study period (4 weeks). Treatment of diabetic rats with *S*. *bachtiarica* extract significantly reduced serum GGT, ALT and AST levels, with comparatively greater activity at a dose of 250 mg/kg. The extract at all doses also ameliorated histological damage of pancreases, liver and kidney tissues. The observed protective effects of *S*. *bachtiarica* extract in diabetic rats are probably related to its antioxidant effects and reduction of STZ-induced oxidative stress as well as reduction of STZ-induced hyperglycaemia.

Treatment of diabetic rats with *S*. *bachtiarica* extract at a dose of 250 mg/kg significantly reduced the expression of *GLUT2* and increased the expression of *GLUT4* in the liver. *GLUT2* is a trans-membrane transporter protein that transports glucose between the liver and blood and plays important role in the hepatic glucose uptake. Unlike *GLUT4*, *GLUT2* does not require insulin for facilitation of glucose transport (Villanueva-Peñacarrillo et al. 2001).

In a previous study, a 2-fold increase in the levels of GLUT2 protein and mRNA has been reported in the liver tissue of STZ-treated diabetic rats, and antidiabetic medications have been shown to lower blood glucose by modulating their levels (Brichard et al. 1993). The GLUT4 isoform is the most important GLUT in muscle and adipose tissues (Kandror and Pilch 1996). Insulin stimulates GLUT4 translocation from the storage vesicles to the plasma membrane and ultimately absorption of glucose in muscle and fat tissues. Resistance to insulin’s stimulating effects on glucose uptake is an important feature of obesity, X syndrome and diabetes mellitus. Up-regulation of *GLUT4* expression and an increase of its translocation to the cell surface considerably increase the permeability of the cell membrane to glucose and improves insulin action on the metabolism of glucose (Villanueva-Peñacarrillo et al. 2001). Some studies have shown that plant polyphenols play an important role in the preventing and managing diabetes by increasing the expression of *GLUT4* in adipose (Jung et al. 2006) and muscle tissues (Zygmunt et al. 2010). As our results indicated, 250 mg/kg dose of *S*. *bachtiarica* extract showed an effective role in the improvement of diabetes-related disorders and also a 1.97-fold increase in *GLUT4* expression and a decrease in *GLUT2* expression in the liver.

## Conclusions

*S*. *bachtiarica* extract has protective effects on hyperglycaemia and diabetes-induced hepatic and renal injury in rats. *S*. *bachtiarica* extract exerts its effects by decreasing the oxidative stress markers and modulating the expression of glucose transporter genes. It is recommended to identify the active chemical constituents of *S*. *bachtiarica* extract, evaluate their effects on animal models of diabetes, and then develop an appropriate clinical trial.
